# Association between pyrethroid exposure and osteoarthritis: a national population-based cross-sectional study in the US

**DOI:** 10.1186/s12889-023-16225-2

**Published:** 2023-08-24

**Authors:** Zhuoshuai Liang, Xiaoyue Sun, Jia Lan, Ruifang Guo, Yuyang Tian, Yawen Liu, Siyu Liu

**Affiliations:** Department of Epidemiology and Biostatistics, School of Public Health of Jilin University, Changchun, 130021 China

**Keywords:** 3-PBA, Pyrethroid, Osteoarthritis, Endocrine disruptor, NHANES

## Abstract

**Background:**

With the restriction of organophosphorus and other insecticides, pyrethroids are currently the second most-used group of insecticides worldwide due to their advantages such as effectiveness and low toxicity for mammalian. Animal studies and clinical case reports have documented associations between adverse health outcomesand exposure to pyrethroids. At present, the association between chronic pyrethroid exposure and osteoarthritis (OA) remains elusive.

**Methods:**

Cross-sectional data from the National Health and Nutrition Examination Survey 1999–2002 and 2007–2014 were used to explore the associations of pyrethroid exposure and OA. Urinary level of 3-phenoxybenzoic acid (3-PBA) in urine samples was used to evaluate the exposure of pyrethroid, and OA was determined on the basis of self-reported physician diagnoses. Multivariable logistic regression models were used to investigate the association between pyrethroid exposure and OA.

**Results:**

Among the 6528 participants, 650 had OA. The weighted geometric mean of urinary volume-based 3-PBA concentration were 0.45 µg/L. With adjustments for major confounders, compared to participants in the lowest quartile of urinary volume-based 3-PBA, those in the highest quartilehad higher odds of OA (odds ratio, 1.39; 95% confidence interval: 1.01, 1.92). There was no nonlinear relationship between urinary volume-based 3-PBA and OA (*P* for non-linearity = 0.89).

**Conclusion:**

High urinary 3-PBA concentration was associated with increased OA odds in the US adults. Pyrethroid exposure in the population should be monitored regularly.

**Supplementary Information:**

The online version contains supplementary material available at 10.1186/s12889-023-16225-2.

## Background

Osteoarthritis (OA), mainly characterized by loss of cartilage and changes of the articular structures, is the most commonly occurring form of arthritis [1, 2]. OA is becoming a worldwide public health concern. Because of the increasing aging population and the increasing rates of obesity, the age-standardized prevalence of OA in the US was increased by 23.2% from 1990 to 2017 [3]. It is estimated that the number of adults with OA in U.S. will increase to 67 million by 2030. Although the complex etiology of OA has not been fully crystallized, the environmental factors are increasingly considered as the causes of the disease [4–6].

Because of advantages such as effectiveness and low toxicity for mammalian, pyrethroids are the second most used pesticides in the world and are widely used for pest control in fabric manufacturing, indoor environments, and agricultural environments, accounting for more than 30% of the global pesticide use [7, 8]. Both dietary and non-dietary factors are important sources for human exposure to pyrethroids [9, 10]. The latest data from the U.S. Food and Drug Administration’s Pesticide Residue Monitoring Program show that pesticide residues were detected in about 47% of domestic food and 49% of imported food samples during examination in 2016 [11]. Dietary determinants of pyrethroid urine metabolite concentrations include fruits and vegetables, poultry and seafood [12]. In addition, potential non-dietary determinants include pyrethroid residues from residential and agricultural applications, which can adhere to dust and soil. Notably, indoor pyrethroid residue concentrations are usually higher than those detected in soil, water, and sediment [13, 14].

Adverse effects of pyrethroids on health outcomes are increasingly documented in recent years [15, 16]. The hormone-like activity of pyrethroid metabolites has been reported to be greater than that of their parent structures, which implies a potential endocrine disrupting effect [17, 18]. Moreover, thyroid hormone and endocrine hormones, such as total testosterone (TT), estrogen, sex hormone-binding globulin (SHBG), are involved in the pathogenesis of OA [19, 20]. Therefore, we hypothesis that pyrethroid exposure is associated with increased OA odds.

To our knowledge, there remains elusive about the association between chronic exposure to pyrethroids and OA. To fill this knowledge gap, we investigated the association of urinary 3-phenoxybenzoic acid (3-PBA), a biomarker for measuring pyrethroids exposure, with OA in US adults: first, we investigated the association of urinary 3-PBA with OA ; second, we examined the nonlinear association of urinary 3-PBA with OA; finally, we explored the association of urinary 3-PBA with OA in subgroups.

## Materials and methods

### Study design and population

The National Health and Nutrition Examination Survey (NHANES) is a cross-sectional survey organized by the National Center for Health Statistics of the Centers for Disease Control and Prevention (CDC). Data of NHANES was obtained using a complex, stratified, clustered multi-staged probability sampling design [21].

A total of 6528 participants aged 20 years and older from six NHANES cycles (1999–2000, 2001–2002, 2007–2008, 2009–2010, 2011–2012, and 2013–2014) were selected in our analyses after excluding 3037 participants with missing information of pyrethroids exposure, OA, sociodemographic, behavioral or health status (Figure S1).

### Pyrethroid exposure assessment

Metabolites of pyrethroid are mainly excreted in urine, thus, pyrethroid exposure is assessed on the basis of concentration of metabolites in urine samples [22]. In NHANES, concentrations of pyrethroid metabolites, including 3-PBA; cis-3-(2,2-dichlorovinyl)-2,2-dimethylcylopropane carboxylic acid; trans-3-(2,2-dichlorovinyl)-2,2-dimethylcylopropane carboxylic acid; 4-fluoro-3-phenoxybenzoic acid ; and cis-(2,2-dibromovinyl)-2,2-dimethylcyclopropane-1-carboxylic acid, were measured in urine samples [15]. We used 3-PBA as a marker of pyrethroid exposure in our analyses, because of a small detected rate of other metabolites of pyrethroids [23]. Notably, the concentration of 3-PBA in urine samples is usually used to evaluate the exposure of pyrethroid pesticides in human biological monitoring studies, and is considered as an acceptable biomarker in epidemiological studies [14, 15, 24]. According to recommendation of NHANES, 3-PBA concentration below the detection limit was replaced by 0.07 µg/L (limit of detection divided by the square root of 2) in our analyses [14, 15].

### Definition of OA

Data of OA status was obtained from questionnaire investigation. Briefly, participants aged 20 years and older were asked “Has a doctor or other health professional ever told you that you have arthritis?”, participants who answered “yes” in this question were then asked “Which type of arthritis was it?”. Participants who answered “osteoarthritis” were classified as having OA, and others were classified as normal [25]. A study showed that agreement between self-reported and clinically confirmed OA has reached 81% [26]. As healthcare becomes increasingly accessible and people pay more attention to health, the consistency between self-reported and clinical diagnosis is likely to improve [27, 28].

### Covariates

Age was regarded as a continuous variable; race/ethnicity was grouped as non-Hispanic Black, Hispanic, non-Hispanic White, and others; education attainment was divided into three categories (less than high school graduate, high school graduate or GED, and some college or above); the family income–to-poverty ratio was defined as the total household income divided by the poverty line and then divided as three level (< 1.30, 1.30–3.49, and ≥ 3.50) [29].

Diet quality was evaluated using the Healthy Eating Index-2015 (HEI-2015), with a total score from 0 to 100, and higher scores indicate higher diet quality. Leisure time physical activity (LTPA) was self-reported and was estimated as minutes of moderate recreational physical activity plus twice the minutes of vigorous recreational physical activity. Based on the 2018 Physical Activity Guidelines for Americans [30], participants with LTPA less than 150 min/wk and with LTPA of 150 min/wk or more were classified as inactive and active, respectively [31]. The body mass index (BMI) was calculated as weight in kilograms divided by height in meters squared. Drinking status was classified as “never”, “former” (a history of daily binge drinking), “current heavy” (≥ 3 drinks per day for females, ≥ 4 drinks per day for males, or binge drinking [≥ 4 drinks on same occasion for females, ≥ 5 drinks on same occasion for males] on 5 or more days per month), “current moderate” (≥ 2 drinks per day for females, ≥ 3 drinks per day for males, or binge drinking ≥ 2 days per month), or “current mild” (current drinking but not meeting the standard of moderate and heavy drinking) [32]. Smoking status was classified as “never” (smoked less than 100 cigarettes in life), “former” (smoked more than 100 cigarettes in life, but they were not currently smoking cigarettes.), or “current smokers” (smoked more than 100 cigarettes in life and smoke some days or every day) [33].

Hypertension was defined as self-reported physician’s diagnosis, use of hypertensive drugs, or blood pressure of ≥ 140/90 mmHg. Diabetes mellitus status (DMs) was classified as “diabetes” (self-reported physician’s diagnosis, a glycosylated hemoglobin [HbA1c] level of ≥ 6.5%, a fasting plasma glucose [FPG] level of ≥ 7.0 mmol/l, random blood glucose level of ≥ 11.1 mmol/l, two-hour glucose tolerance test blood glucose level of ≥ 11.1 mmol/l, or use of diabetes medication or insulin), “prediabetes” (FPG of 5.6-7.0 mmol/l, 2-h plasma glucose of 7.8–11.0 mmol/l, HbA1c of 5.7–6.4%, or self-reported), or “normal” [34].

### Statistical analysis

We used survey-specific sample weights to account for unequal selection probabilities, oversampling of certain subgroups, and nonresponse adjustment to ensure nationally representative estimates. According to NHANES Analytic and Reporting Guidelines, we incorporated the sample weights for 1999–2002 and 2007–2014 [35].

Geometric mean and its 95% confidence interval (CI) were shown for volume-based and creatinine-corrected urinary 3-PBA concentrations, respectively. Continuous variables were expressed as weighted mean with CIs, and categorical variables were expressed as weighted frequency distribution. Baseline characteristics were compared using the survey-weighted linear regression for continuous variables and the survey-weighted Chi-square test for categorical variables.

Notably, concentrations of creatinine may vary with age, sex, and race/ethnicity. For a diversified population from NHANES, it may not be optimal to use the creatinine-correction value of urinary 3-PBA concentration for analysis [15]. Therefore, we used volume-based 3-PBA concentration (unadjusted for creatinine) in the analysis with urinary creatinine added as a separate independent variable [15, 36]. In addition, we used creatinine-corrected 3-PBA as the primary exposure in sensitivity analyses to test the robustness of identified associations.

Urinary 3-PBA was modeled both as a continuous variable with log-transformation, and as quartiles with the lowest quartile regarded as the reference group. Three multivariate logistic regression models were constructed to estimate the odds ratios (ORs) and 95% CIs for associations of 3-PBA and OA. Model 1 was adjusted for urinary creatinine only; model 2 was additionally adjusted for age, gender, race/ethnicity, family poverty-to-income ratio, and education attainment; model 3 was additionally adjusted for diet quality (HEI-2015 score), LTPA, smoking status, drinking status, BMI, DMs, and hypertension.

Linear trends (dose-response relationships) were estimated by assigning the median 3-PBA value for each 3-PBA quartiles as a continuous variable. Moreover, logistic regression based on restricted cubic splines with three knots was conducted to examine the nonlinear association between 3-PBA and OA.

Finally, subgroup analyses were performed by sex, age (< 50 and ≥ 50), race/ethnicity, BMI (< 25 and ≥ 25), LTPA, DMs, and hypertension. All analyses were conducted using R software 4.2.1, and a two-tailed *P* value < 0.05 was considered statistically significant.

## Results

### Basic characteristics of study participants

Among the 6528 participants, 650 had OA. The weighted geometric mean (95% CI) of volume-based and creatinine-corrected urinary 3-PBA concentrations were 0.45 µg/L (95% CI: 0.43, 0.48) and 0.47 µg/g (95% CI: 0.44, 0.50), respectively. Volume-based urinary 3-PBA concentrations were higher in non-Hispanic blacks, current smokers than their counterparts, and creatinine-corrected 3-PBA concentrations were higher in non-Hispanic whites, current or former smokers, and those over 50 years than their counterparts (Table S1). Compared with participants without OA, those with OA are prone to female, non-Hispanic white, having higher family income–to-poverty ratio, former smoking, former drinking, having hypertension, and having DM. Notably, the levels both of urinary volume-based 3-PBA and urinary creatinine-corrected 3-PBA in participants with OA were significantly higher than those in participants without OA (Table 1). The details of quartiles of the 3-PBA level are shown in Table 2.

**Table 1:** Baseline characteristics of study participants (N = 6528)

Characteristics	Non-OA (n = 5878)	OA (n = 650)	*P*-value
**Age years, mean**	42.69	59.72	**< 0.01**
**Urinary volume-based 3-PBA, n (%)**			**0.04**
Q1	1475 (26.14)	140 (22.73)	
Q2	1416 (24.82)	156 (26.23)	
Q3	1498 (24.73)	153 (21.15)	
Q4	1487 (24.31)	201 (29.90)	
**Urinary creatinine-corrected 3-PBA, n (%)**			**< 0.01**
Q1	1523 (26.36)	117 (18.01)	
Q2	1485 (25.15)	145 (22.98)	
Q3	1448 (24.43)	185 (26.57)	
Q4	1420 (24.05)	203 (32.44)	
**Sex, n (%)**			**< 0.01**
Male	3054 (51.51)	220 (31.33)	
Female	2824 (48.49)	430 (68.67)	
**Race/ethnicity, n (%)**			**< 0.01**
Hispanic	1549 (14.49)	91 (4.63)	
Non-Hispanic black	1172 (10.56)	87 (5.89)	
Non-Hispanic white	2644 (68.46)	436 (84.44)	
Others race	513 (6.50)	36 (5.04)	
**Education, n (%)**			0.31
Less than high school graduate	1418 (16.08)	125 (13.56)	
High school graduate or GED	1297 (21.56)	140 (20.56)	
Some college or above	3163 (62.36)	385 (65.87)	
**Family income–to-poverty ratio**			**< 0.01**
< 1.30	1829 (21.73)	164 (15.12)	
1.30–3.49	2134 (33.91)	258 (38.43)	
≥ 3.50	1915 (44.36)	228 (46.45)	
**BMI (kg/m** ^**2**^ **)**			**< 0.01**
< 25	1910 (93.23)	132 (6.77)	
≥ 25	3968 (87.80)	518 (12.20)	
**Smoking status, n (%)**			**< 0.01**
Never	3245 (55.59)	309 (48.53)	
Former	1290 (21.77)	231 (36.13)	
Current	1343 (22.64)	110 (15.34)	
**Alcohol, n (%)**			**< 0.01**
Never	800 (11.51)	95 (13.08)	
Former	959 (13.53)	156 (21.88)	
Mild	1839 (34.32)	239 (38.72)	
Moderate	942 (16.98)	97 (15.51)	
Heavy	1338 (23.66)	63 (10.81)	
**LTPA, n (%)**			0.13
Inactive	2654 (43.84)	339 (47.38)	
Active	3224 (56.16)	311 (52.62)	
**Hypertension, n (%)**			**< 0.01**
No	3828 (70.14)	221 (39.69)	
Yes	2050 (29.86)	429 (60.31)	
**DMs, n (%)**			**< 0.01**
Normal	4699 (84.67)	431 (72.39)	
Prediabetes	412 (6.19)	62 (9.15)	
Diabetes	765 (9.14)	157 (18.46)	

The bold values mean statistical significance

Abbreviations: OA, osteoarthritis; 3-PBA, 3-phenoxybenzoic acid; LTPA, leisure-time physical activity; Q, quartile; BMI, body mass index; DMs, diabetes mellitus status; 3-PBA, 3-phenoxybenzoic acid

**Table 2:** Details of quartiles of the 3-PBA level

3-PBA	Geometric mean (CI)	Median	Interquartile range
Urinary volume-based, µg/L			
Q1	0.08 (0.08, 0.08)	0.07	(0.07, 0.07)
Q2	0.27 (0.26, 0.27)	0.28	(0.21, 0,35)
Q3	0.68 (0.67, 0.69)	0.68	(0.54, 0.85)
Q4	3.24 (3.04, 3.45)	2.45	(1.54, 5.51)
Urinary creatinine-corrected, µg/g			
Q1	0.09 (0.09, 0.10)	0.10	(0.06, 0.15)
Q2	0.30 (0.30, 0.30)	0.30	(0.25, 0.36)
Q3	0.63 (0.62, 0.64)	0.63	(0.51, 0.78)
Q4	2.93 (2.77, 3.09)	2.29	(1.44, 5.04)

Abbreviations: 3-PBA, 3-phenoxybenzoic acid; CI, confidence interval; Q, quartile

### Associations between 3-PBA and OA

**Fig. 1:**
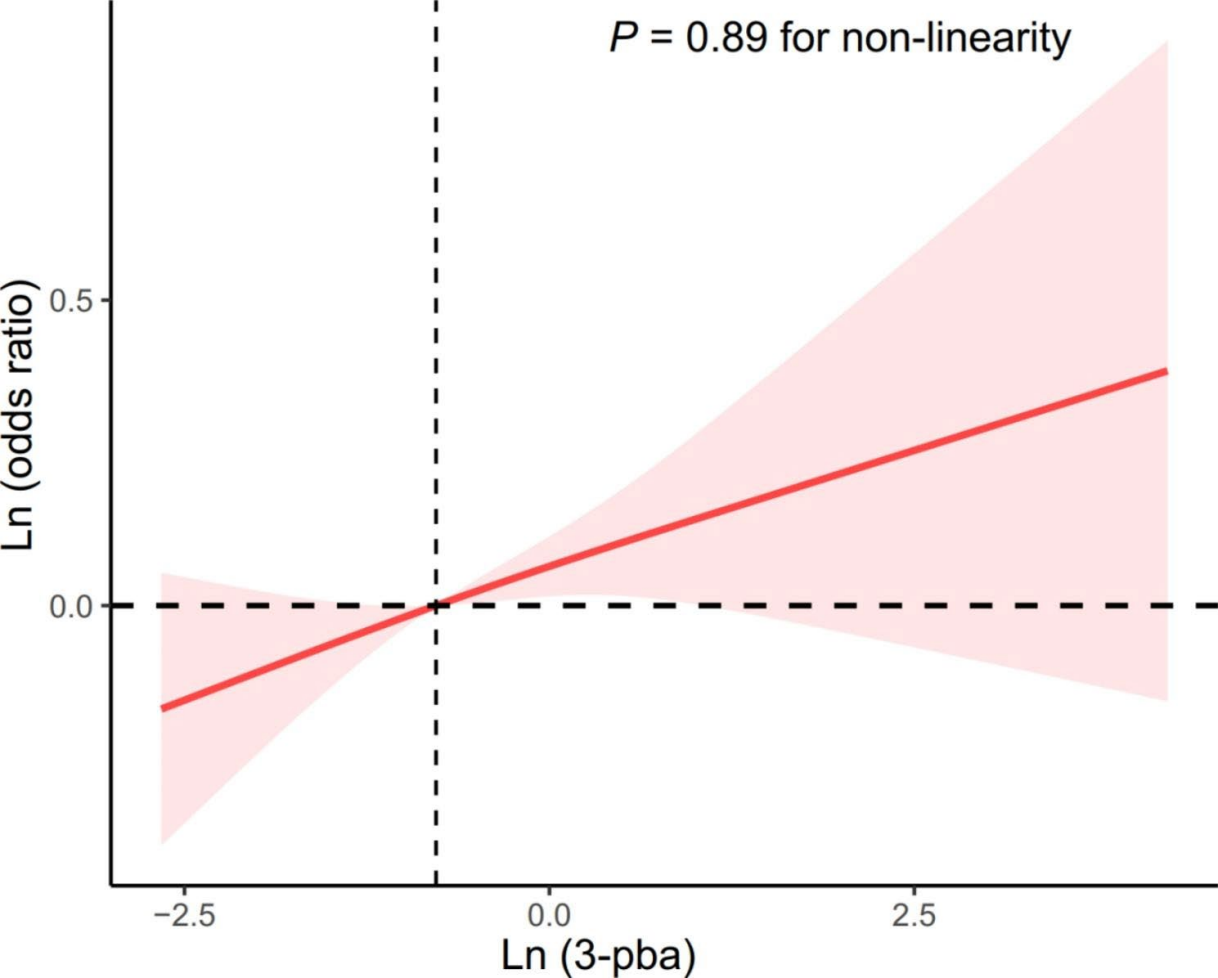
Dose-response association between urinary volume-based 3-PBA and OA Note: There exist linear associations between urinary volume-based 3-PBA and OA (*P* = 0.89 for non-linearity). The red lines and shaded areas represent the hazard ratios estimates and 95% CIs, respectively, relative to the reference level (dotted vertical lines). Models were adjusted for urinary creatinine, age, sex, race/ethnicity, education, family poverty-to-income ratio, HEI-2015, LTPA, smoking status, drink status, BMI, DMs and hypertension. Abbreviations: 3-PBA, 3-phenoxybenzoic acid; OA, osteoarthritis

The OR of OA was positively correlated with urinary volume-based 3-PBA concentrations in all models (Table 3). Each unit increase in ln (urinary volume-based 3-PBA) was associated with 9% increased OA odds. Furthermore, compared with participants in the lowest quartile, those with the highest quartile of urinary volume-based 3-PBA had higher odds of OA (OR, 1.39; 95% CI: 1.00, 1.92) (*P* for trend = 0.07). Regressions based on restricted cubic splines showed that there was no nonlinear relationship between urinary volume-based 3-PBA and OA (*P* for non-linearity = 0.89) (Fig. 1).

**Table 3:** OR (95% CIs for association between urinary volume-based 3-PBA and OA

Models	Urinary volume-based3-PBA, µg/L					*P* for trend
	Ln	Q1	Q2	Q3	Q4	
**OA**						0.07
Model 1	**1.17** **(1.10, 1.25)**	1.00 (ref)	1.31 (0.96, 1.80)	1.25 (0.93, 1.68)	**1.87** **(1.41, 2.48)**	
Model 2	**1.09** **(1.01, 1.17)**	1.00 (ref)	1.32 (0.95, 1.83)	1.07 (0.76, 1.49)	**1.41** **(1.03, 1.92)**	
Model 3	**1.09** **(1.01, 1.17)**	1.00 (ref)	1.26 (0.90, 1.77)	1.00 (0.71, 1.40)	**1.39** **(1.00, 1.92)**	

The bold values mean statistical significance

Model 1: Adjusted for urinary creatinine (continuous, mg/dL)

Model 2: Adjusted for age (continuous, years), sex (male, female), race/ethnicity (Hispanic, non-Hispanic white, non-Hispanic black, and others), education (less than high school, high school and college or higher), and family poverty-to-income ratio (< 1.3, 1.3–3.5, and > 3.5) plus variables in Model 1

Model 3: Adjusted for diet quality (continuous, HEI-2015 score), LTPA (inactive and active), smoking status (never, former, and current), drinking status (never, former, mild, moderate, and heavy), BMI, DMs (diabetes, prediabetes, and normal) and hypertension (yes and no) plus variables in Model 2

Abbreviation: OR, odds ratio; CIs, confidence intervals; OA, osteoarthritis; 3-PBA, 3-phenoxybenzoic acid; Q, quartile

### Subgroup analysis

Subgroup analysis showed a statistically significant positive association between urinary volume-based 3-PBA and OA in female (OR, 1.16; 95% CI: 1.05, 1.27, *P* for trend < 0.01), non-Hispanic whites (OR, 1.11; 95% CI: 1.02, 1.20, *P* for trend = 0.04), participants with BMI ≥ 25 (OR, 1.10; 95% CI: 1.01, 1.20, *P* for trend = 0.05), participants with actively LTPA (OR, 1.15; 95% CI: 1.00, 1.32, *P* for trend = 0.03), and participants without diabetes or prediabetes (OR, 1.13; 95% CI: 1.03, 1.24, *P* for trend = 0.02) (Table 4). However, only sex interacted with urinary volume-based 3-PBA for OA (*P* for interaction = 0.03).

**Table 4:** OR (95% CIs) for association between urinary volume-based 3-PBA and OA in subgroups

Group	Ln	Q1	Q2	Q3	Q4	*P* for trend	*P* for interaction
Sex							**0.03**
Male	0.98 (0.86, 1.13)	1.00 (ref)	1.12 (0.69, 1.84)	0.68 (0.40, 1.17)	0.85 (0.51, 1.41)	0.44	
Female	**1.16 (1.05, 1.27)**	1.00 (ref)	1.37 (0.90, 2.10)	1.27 (0.82, 1.97)	**1.90 (1.26, 2.86)**	**< 0.01**	
Age							0.96
< 50	1.08 (0.89, 1.29)	1.00 (ref)	1.12 (0.55, 2.28)	0.97 (0.54, 1.76)	1.34 (0.69, 2.59)	0.38	
≥ 50	1.09 (0.99, 1.20)	1.00 (ref)	1.35 (0.92, 1.98)	1.11 (0.73, 1.69)	**1.48 (1.01, 2.19)**	0.10	
Race/ethnicity							0.12
Hispanic	1.02 (0.86, 1.21)	1.00 (ref)	1.33 (0.62, 2.83)	1.61 (0.79, 3.29)	1.73 (0.75, 3.96)	0.32	
Non-Hispanic white	**1.11 (1.02, 1.20)**	1.00 (ref)	1.37 (0.93, 2.00)	1.04 (0.69, 1.55)	**1.54 (1.08, 2.18)**	**0.04**	
Non-Hispanic black	1.19 (0.98, 1.43)	1.00 (ref)	2.07 (0.81, 5.25)	1.75 (0.73, 4.24)	1.91 (0.82, 4.46)	0.45	
Others Race	0.67 (0.42, 1.07)	1.00 (ref)	0.44 (0.12, 1.61)	**0.20 (0.04, 0.97)**	**0.15 (0.03, 0.88)**	0.09	
BMI							0.60
< 25	1.03 (0.87, 1.22)	1.00 (ref)	0.96 (0.42, 2.20)	1.09 (0.57, 2.10)	1.18 (0.61, 2.29)	0.57	
≥ 25	**1.10 (1.01, 1.20)**	1.00 (ref)	1.37 (0.99, 1.91)	1.03 (0.69, 1.53)	**1.49 (1.03, 2.15)**	0.05	
LTPA							0.47
Active	**1.15 (1.00, 1.32)**	1.00 (ref)	1.74 (0.96, 3.16)	1.23 (0.69, 2.18)	**2.26 (1.30, 3.95)**	**0.03**	
Inactive	1.07 (0.98, 1.17)	1.00 (ref)	1.15 (0.80, 1.63)	0.99 (0.67, 1.45)	1.20 (0.82, 1.75)	0.41	
DMs							0.12
Normal	**1.13 (1.03, 1.24)**	1.00 (ref)	1.43 (0.96, 2.13)	0.99 (0.64, 1.54)	**1.69 (1.18, 2.44)**	**0.02**	
Prediabetes	1.09 (0.88, 1.36)	1.00 (ref)	0.61 (0.20, 1.82)	1.39 (0.47, 4.09)	1.10 (0.42, 2.94)	0.65	
Diabetes	0.92 (0.76, 1.12)	1.00 (ref)	1.01 (0.48, 2.10)	0.83 (0.38, 1.86)	0.72 (0.31, 1.69)	0.34	
Hypertension							0.85
Yes	1.10 (0.99, 1.22)	1.00 (ref)	1.28 (0.85, 1.94)	0.92 (0.60, 1.39)	1.39 (0.89, 2.16)	0.18	
No	1.06 (0.94, 1.19)	1.00 (ref)	1.22 (0.73, 2.03)	1.10 (0.66, 1.83)	1.35 (0.83, 2.18)	0.32	

All models were adjusted for urinary creatinine, age, sex, race/ethnicity, education, family poverty-to-income ratio, HEI-2015, LTPA, smoking status, drink status, BMI, DMs and hypertension

The bold values mean statistical significance

Abbreviation: OR, odds ratio; CIs, confidence intervals; OA, osteoarthritis; 3-PBA, 3-phenoxybenzoic acid; Q, quartile; BMI, body mass index; LTPA, leisure-time physical activity; DMs, diabetes mellitus status

### Sensitivity analysis

To examine the robustness of association between urinary 3-PBA concentration and OA, we regarded urinary creatinine-corrected 3-PBA as the primary exposure and conducted same analyses as volume-based 3-PBA. As expected, we got consistent results (Table S2, Figure S2, and Table S3).

## Discussion

In this nationally representative sample of US adults, we documented that increased levels of urinary 3-PBA concentrations were associated with increased OA odds. Moreover, these association remained consistent in sensitivity analyses and in most subgroups.

Different availability of pyrethroid-contained products or environments may confer the different pyrethroids exposure for population in different country. In this study, we found that the geometric mean of creatinine-corrected 3-PBA concentration was 0.47 µg/g creatine in US adults. It was lower than reported in China (0.93 µg/g) [37] and South Korea (1.95 µg/g) [38], but was higher than that in Japan (0.40 µg/g) [39]. Notably, an increasing trend of urinary 3-PBA levels in US children and adults was documented [8], suggesting that exposure of pyrethroid has been an urgent public health issue.

3-PBA has anti-thyroid hormone activity and exhibits strong thyroid hormone receptor antagonistic capacity [40]. Long-term pyrethroid exposure in mice affects levels of thyroxine and triiodothyronine in the serum and brain tissue [41]. Furthermore, population study has documented that high urinary 3-PBA levels are associated with the decrease of thyroid hormones level [38]. Notably, thyroid hormones play a key role in the development of OA through stimulating terminal chondrocyte differentiation and transglutaminase activity in articular cartilage [42, 43]. Therefore, pyrethroids exposure with anti-thyroid hormone activity may exert a prominent impact on development of OA.

Pyrethroids can also affect sex hormone. Pyrethroids and their metabolites showed anti-estrogenic activity in human and rat estrogen receptor α [18, 44], which may lead to type II collagen degradation and related structural alterations [45]. Moreover, high urinary 3-PBA is associated with increased SHBG and TT, and levels of TT and SHBG are positively associated with OA risk [46, 47]. Therefore, we reasonably speculate that 3-PBA increases the odds of OA through affecting thyroid hormone and sex hormone levels in the body.

At present, the risk factors confirmed for OA including increase of age, genetics, obesity, females, and exercise [5, 6]. Interestingly, we found that 3-PBA was significantly associated with the odds of OA in these corresponding subgroups, indicating that pyrethroids or their metabolites may interact with these risk factors for OA. As shown in our subgroup results, we observed a significant interaction between sex and 3-PBA for OA, indicating a stronger endocrine disruptor activity of pyrethroids in female than in male. Indeed, female are at higher risk of OA than male, which may be conferred by difference of genetics and anatomic. Heritability of OA is stronger in female than in male [48], moreover, the thickness of articular cartilage of distal femur and patella in female is lower than that in male, and femurs in female are narrower than those in male [48]. In addition, changes in hormone levels may mediate age–sex interaction in OA risk. For example, hormone levels in the body changes greatly after menopause, leading to an increased OA risk [47]; causal relationships of TT and SHBG with OA were more likely to be observed in female than in male [46, 47]. Similarly, association between 3-PBA and TT varies by sex, that a positive association among female but no association among male [49]. Moreover, TT is mainly secreted by testes in male, while TT is directly secreted by adrenal gland or ovary, or is indirectly transformed from testosterone precursor in female [49, 50]. In this case, pyrethroids and their metabolites may target different action pathways for female and male, leading to heterogeneity of association between 3-PBA and OA. However, the potential relationship between pyrethroids and OA remains to be explored.

There existed heterogeneity of association among others subgroups, including age, race, BMI, LTPA, and DMs. In addition to the lower power of test caused by small samples, several biological and behavioral factors can partly explain this heterogeneity. We observed a significant positive association between urinary 3-PBA and OA odds in overweight or obese participants rather than in normal weight participants. One reasonable explanation may be that extra mass increases the joint load and causes additional stress on the articular cartilage, thus leading to degenerative changes in the weight bearing joints and OA susceptibility [51]. Another explanation may be that effect of endocrine disrupting chemicals may be modified by metabolic changes caused by fat distribution, such as modulations in insulin resistance or leptin production [14]. Interestingly, we found that participants with actively LTPA seem to be more susceptible to the effect of pyrethroid exposure. Indeed, the relationship between physical activity and OA is complex. Although the role of physical activity in the treatment of OA has been highlighted [52], high intensity physical activity seems to increase the odds of OA, and it is unclear whether this association is due to physical activity or injury [6]. The potential modifying effect of physical exercise on association between pyrethroid exposure and OA needs to be further clarified.

Some strengths in this study must be underscored. We provided novel insights into the association between pyrethroid exposure and health outcomes. Moreover, we considered a wide range of potential confounders including socioeconomic status, dietary, lifestyle, and comorbidities for analysis. Limitations should be acknowledged for interpreting our findings. Firstly, the identified association between pyrethroid exposure and OA may not be causal because of a cross-sectional design; secondly, the data of 3-PBA in the NHANES comes from one single spot urine samples, and the short half-life of pyrethroids in humans may damage the credibility of 3-PBA as a reflection of long-term exposure status [53]; finally, the assessment of OA was collected by questionnaire survey, thereby increasing the risk of measurement bias.

## Conclusion

Urinary 3-PBA concentration was positively associated with OA odds in the US adults. Our study highlights for the first time the association between chronic exposure to pyrethroids and OA. Pyrethroid exposure in the population should be monitored regularly.

### Electronic supplementary material

Below is the link to the electronic supplementary material.

Supplementary Material 1

## Data Availability

This study utilized publicly available data from the National Health and Nutrition Examination Survey. This data can be accessed using the following link: https://www.cdc.gov/nchs/nhanes/index.htm.

## Abbreviations

OA: Osteoarthritis
TT: Total testosterone
3-PBA: 3-phenoxybenzoic acid
NHANES: National Health and Nutrition Examination Survey
CDC: Centers for Disease Control and Prevention
BMI: Body mass index
HbA1c: Glycosylated hemoglobin
FPG: Fasting plasma glucose
CI: Confidence interval
ORs: Odds ratios
HEI-2015: Healthy Eating Index-2015
DMs: Diabetes mellitus status
LTPA: Leisure time physical activity

## References

[1] Glyn-Jones S, Palmer AJ, Agricola R, Price AJ, Vincent TL, Weinans H, Carr AJ (2015). Osteoarthritis. Lancet.

[2] Martel-Pelletier J, Barr AJ, Cicuttini FM, Conaghan PG, Cooper C, Goldring MB, Goldring SR, Jones G, Teichtahl AJ, Pelletier JP (2016). Osteoarthritis. Nat Rev Dis Primers.

[3] Safiri S, Kolahi AA, Smith E, Hill C, Bettampadi D, Mansournia MA, Hoy D, Ashrafi-Asgarabad A, Sepidarkish M, Almasi-Hashiani A (2020). Global, regional and national burden of osteoarthritis 1990–2017: a systematic analysis of the global burden of Disease Study 2017. Ann Rheum Dis.

[4] Chen L, Zhao Y, Liu F, Chen H, Tan T, Yao P, Tang Y (2022). Biological aging mediates the associations between urinary metals and osteoarthritis among U.S. adults. BMC Med.

[5] Prieto-Alhambra D, Judge A, Javaid MK, Cooper C, Diez-Perez A, Arden NK (2014). Incidence and risk factors for clinically diagnosed knee, hip and hand osteoarthritis: influences of age, gender and osteoarthritis affecting other joints. Ann Rheum Dis.

[6] Palazzo C, Nguyen C, Lefevre-Colau MM, Rannou F, Poiraudeau S (2016). Risk factors and burden of osteoarthritis. Annals of physical and rehabilitation medicine.

[7] Saillenfait AM, Ndiaye D, Sabaté JP (2015). Pyrethroids: exposure and health effects–an update. Int J Hyg Environ Health.

[8] Lehmler HJ, Simonsen D, Liu B, Bao W (2020). Environmental exposure to pyrethroid pesticides in a nationally representative sample of U.S. adults and children: the National Health and Nutrition Examination Survey 2007–2012. Environ pollution (Barking Essex: 1987).

[9] Rodzaj W, Wileńska M, Klimowska A, Dziewirska E, Jurewicz J, Walczak-Jędrzejowska R, Słowikowska-Hilczer J, Hanke W, Wielgomas B (2021). Concentrations of urinary biomarkers and predictors of exposure to pyrethroid insecticides in young, polish, urban-dwelling men. Sci Total Environ.

[10] Balalian AA, Liu X, Siegel EL, Herbstman JB, Rauh V, Wapner R, Factor-Litvak P, Whyatt R. Predictors of urinary pyrethroid and organophosphate compound concentrations among healthy pregnant women in New York. Int J Environ Res Public Health 2020, 17(17). 10.3390/ijerph17176164 PMC7504694 32854291

[11] Hyland C, Bradman A, Gerona R, Patton S, Zakharevich I, Gunier RB, Klein K (2019). Organic diet intervention significantly reduces urinary pesticide levels in U.S. children and adults. Environ Res.

[12] Glorennec P, Serrano T, Fravallo M, Warembourg C, Monfort C, Cordier S, Viel JF, Le Gléau F, Le Bot B, Chevrier C (2017). Determinants of children’s exposure to pyrethroid insecticides in western France. Environ Int.

[13] Werthmann DW, Rabito FA, Stout DM 2nd, Tulve NS, Adamkiewicz G, Calafat AM, Ospina M, Chew GL. Pyrethroid exposure among children residing in green versus non-green multi-family, low-income housing. J Expo Sci Environ Epidemiol. 2021;31(3):549–59. 10.1038/s41370-021-00312-w PMC8140995 33677471

[14] Guo X, Li N, Wang H, Su W, Song Q, Liang Q, Sun C, Liang M, Ding X, Lowe S et al. Exploratory analysis of the association between pyrethroid exposure and rheumatoid arthritis among US adults: 2007–2014 data analysis from the National Health and Nutrition Examination Survey (NHANES). *Environmental science and pollution research international* 2022. 10.1007/s11356-022-23145-y 36151437

[15] Xue Q, Pan A, Wen Y, Huang Y, Chen D, Yang CX, Hy Wu J, Yang J, Pan J, Pan XF (2021). Association between pyrethroid exposure and cardiovascular disease: a national population-based cross-sectional study in the US. Environ Int.

[16] Xu H, Mao Y, Xu B (2020). Association between pyrethroid pesticide exposure and hearing loss in adolescents. Environ Res.

[17] Brander SM, Gabler MK, Fowler NL, Connon RE, Schlenk D (2016). Pyrethroid pesticides as endocrine disruptors: Molecular Mechanisms in vertebrates with a focus on fishes. Environ Sci Technol.

[18] Saillenfait AM, Ndiaye D, Sabaté JP (2016). The estrogenic and androgenic potential of pyrethroids in vitro. Rev Toxicol vitro: Int J published association BIBRA.

[19] Bassett JD, Williams GRJEr. Role of thyroid hormones in skeletal development and bone maintenance. 2016, 37(2):135–87. 10.1210/er.2015-1106 PMC4823381 26862888

[20] Bay-Jensen AC, Slagboom E, Chen-An P, Alexandersen P, Qvist P, Christiansen C, Meulenbelt I, Karsdal MA (2013). Role of hormones in cartilage and joint metabolism: understanding an unhealthy metabolic phenotype in osteoarthritis. Menopause (New York NY).

[21] Chen T-C, Clark J, Riddles MK, Mohadjer LK, Fakhouri TH. National Health and Nutrition Examination Survey, 2015 – 2018: sample design and estimation procedures. 2020. 33663649

[22] Klimowska A, Amenda K, Rodzaj W, Wileńska M, Jurewicz J, Wielgomas B (2020). Evaluation of 1-year urinary excretion of eight metabolites of synthetic pyrethroids, chlorpyrifos, and neonicotinoids. Environ Int.

[23] Barr DB, Olsson AO, Wong LY, Udunka S, Baker SE, Whitehead RD, Magsumbol MS, Williams BL, Needham LL (2010). Urinary concentrations of metabolites of pyrethroid insecticides in the general U.S. population: National Health and Nutrition Examination Survey 1999–2002. Environ Health Perspect.

[24] Wielgomas B (2013). Variability of urinary excretion of pyrethroid metabolites in seven persons over seven consecutive days–implications for observational studies. Toxicol Lett.

[25] Li Y, Zhu J, Fan J, Cai S, Fan C, Zhong Y, Sun L (2020). Associations of urinary levels of phenols and parabens with osteoarthritis among US adults in NHANES 2005–2014. Ecotoxicol Environ Saf.

[26] March LM, Schwarz JM, Carfrae BH, Bagge E (1998). Clinical validation of self-reported osteoarthritis. Osteoarthr Cartil.

[27] Selçuk H, Roos EM, Grønne DT, Ernst MT, Skou ST (2021). Agreement between Self-Reported information and administrative data on Comorbidities, Imaging and Treatment in Denmark - A Validation Study of 38,745 patients with knee or hip osteoarthritis. Clin Epidemiol.

[28] Davis AM, King LK, Stanaitis I, Hawker GA (2022). Fundamentals of osteoarthritis: outcome evaluation with patient-reported measures and functional tests. Osteoarthr Cartil.

[29] Vilar-Gomez E, Nephew LD, Vuppalanchi R, Gawrieh S, Mladenovic A, Pike F, Samala N, Chalasani N (2022). High-quality diet, physical activity, and college education are associated with low risk of NAFLD among the US population. Hepatology (Baltimore MD).

[30] Piercy KL, Troiano RP, Ballard RM, Carlson SA, Fulton JE, Galuska DA, George SM, Olson RD (2018). The physical activity guidelines for Americans. JAMA.

[31] Ussery EN, Fulton JE, Galuska DA, Katzmarzyk PT, Carlson SA (2018). Joint prevalence of sitting time and leisure-time physical activity among US adults, 2015–2016. JAMA.

[32] Rattan P, Penrice DD, Ahn JC, Ferrer A, Patnaik M, Shah VH, Kamath PS, Mangaonkar AA, Simonetto DA (2022). Inverse Association of Telomere length with Liver Disease and Mortality in the US Population. Hepatol Commun.

[33] Natto SSYAL, Midle ZS, Gyurko JB, O’Neill R, Steffensen R (2019). Association between time since quitting smoking and periodontitis in former smokers in the National Health and Nutrition examination surveys (NHANES) 2009 to 2012. J Periodontol.

[34] Chen C, Chen Q, Nie B, Zhang H, Zhai H, Zhao L, Xia P, Lu Y, Wang N (2020). Trends in Bone Mineral density, osteoporosis, and Osteopenia among U.S. adults with Prediabetes, 2005–2014. Diabetes Care.

[35] Prevention, CfDCa. *NHANES Survey Methods and Analytic Guidelines* Accessed 31 December 2022.

[36] Barr DB, Wilder LC, Caudill SP, Gonzalez AJ, Needham LL, Pirkle JL (2005). Urinary creatinine concentrations in the U.S. population: implications for urinary biologic monitoring measurements. Environ Health Perspect.

[37] Han Y, Xia Y, Han J, Zhou J, Wang S, Zhu P, Zhao R, Jin N, Song L, Wang X (2008). The relationship of 3-PBA pyrethroids metabolite and male reproductive hormones among non-occupational exposure males. Chemosphere.

[38] Hwang M, Lee Y, Choi K, Park C (2019). Urinary 3-phenoxybenzoic acid levels and the association with thyroid hormones in adults: Korean National Environmental Health Survey 2012–2014. Sci Total Environ.

[39] Ueyama J, Kimata A, Kamijima M, Hamajima N, Ito Y, Suzuki K, Inoue T, Yamamoto K, Takagi K, Saito I (2009). Urinary excretion of 3-phenoxybenzoic acid in middle-aged and elderly general population of Japan. Environ Res.

[40] Du G, Shen O, Sun H, Fei J, Lu C, Song L, Xia Y, Wang S, Wang X (2010). Assessing hormone receptor activities of pyrethroid insecticides and their metabolites in reporter gene assays. Toxicol Sci.

[41] Wang S, Shi N, Ji Z, Pinna G (2002). [Effects of pyrethroids on the concentrations of thyroid hormones in the rat serum and brain]. Zhonghua Lao Dong Wei Sheng Zhi Ye Bing Za Zhi.

[42] Randau TM, Schildberg FA, Alini M, Wimmer MD, Haddouti el M, Gravius S, Ito K, Stoddart MJ (2013). The effect of dexamethasone and triiodothyronine on terminal differentiation of primary bovine chondrocytes and chondrogenically differentiated mesenchymal stem cells. PLoS ONE.

[43] Rosenthal AK, Heinkel D, Gohr CM (2003). Thyroxine stimulates transglutaminase activity in articular chondrocytes. Osteoarthr Cartil.

[44] Sun H, Chen W, Xu X, Ding Z, Chen X, Wang X (2014). Pyrethroid and their metabolite, 3-phenoxybenzoic acid showed similar (anti)estrogenic activity in human and rat estrogen receptor α-mediated reporter gene assays. Environ Toxicol Pharmacol.

[45] Oestergaard S, Sondergaard BC, Hoegh-Andersen P, Henriksen K, Qvist P, Christiansen C, Tankó LB, Karsdal MA (2006). Effects of ovariectomy and estrogen therapy on type II collagen degradation and structural integrity of articular cartilage in rats: implications of the time of initiation. Arthritis Rheum.

[46] Qu Z, Huang J, Yang F, Hong J, Wang W, Yan S (2020). Sex hormone-binding globulin and arthritis: a mendelian randomization study. Arthritis Res therapy.

[47] Yan YS, Qu Z, Yu DQ, Wang W, Yan S, Huang HF (2021). Sex steroids and osteoarthritis: a mendelian randomization study. Front Endocrinol.

[48] O’Connor MI (2007). Sex differences in osteoarthritis of the hip and knee. J Am Acad Orthop Surg.

[49] Xu H, Bo Y (2022). Associations between pyrethroid exposure and serum sex steroid hormones in adults: findings from a nationally representative sample. Chemosphere.

[50] Jaspers L, Dhana K, Muka T, Meun C, Kiefte-de Jong JC, Hofman A, Laven JS, Franco OH, Kavousi M (2016). Sex steroids, sex hormone-binding globulin and Cardiovascular Health in Men and Postmenopausal Women: the Rotterdam Study. J Clin Endocrinol Metab.

[51] Kulkarni K, Karssiens T, Kumar V, Pandit H (2016). Obesity and osteoarthritis. Maturitas.

[52] Gay C, Chabaud A, Guilley E, Coudeyre E (2016). Educating patients about the benefits of physical activity and exercise for their hip and knee osteoarthritis. Systematic literature review. Annals of physical and rehabilitation medicine.

[53] Appenzeller BMR, Hardy EM, Grova N, Chata C, Faÿs F, Briand O, Schroeder H, Duca RC (2017). Hair analysis for the biomonitoring of pesticide exposure: comparison with blood and urine in a rat model. Arch Toxicol.

