# Supplementary material for: Association between pyrethroid exposure and osteoarthritis: a national population-based cross-sectional study in the US
Source: BMC Public Health. 2023 Aug 24;23:1521. doi: 10.1186/s12889-023-16225-2 (PMC10464395; doi:10.1186/s12889-023-16225-2)
Supplement: Supplementary file 1 — Supplementary Material 1 [file 12889_2023_16225_MOESM1_ESM.docx]

**Association between pyrethroid exposure and osteoarthritis: A national population-based cross-sectional study in the US**

Zhuoshuai Liang^1#^, Xiaoyue Sun^1#^, Jia Lan^1^, Ruifang Guo^1^, Yuyang Tian^1^, Yawen Liu^1^*, Siyu Liu^1^*

^1^ Department of Epidemiology and Biostatistics, School of Public Health of Jilin University, Changchun 130021, China.

^#^ These authors contributed equally to this work.

* Correspondence to: Siyu Liu, PhD; Yawen Liu, PhD

Email: liusiyu@jlu.edu.cn (Siyu Liu); ywliu@jlu.edu.cn (Yawen Liu)

Tel: 86-431-85619437 (Yawen Liu)

Journal: [*BMC*](https://www.letpub.com.cn/index.php?page=journalapp&view=detail&journalid=2567) *Public Health*

Table S1 Baseline characteristics of participants (N =6528).

| Characteristics | Total | Urinary volume-based 3-PBA, μg/L | |  | Urinary creatinine-  corrected 3-PBA, μg/g | |
| --- | --- | --- | --- | --- | --- | --- |
|  |  | Geometric  mean  (95% CI) | *P*-value |  | Geometric  mean  (95% CI) | *P*-value |
| Total | 6528 | 0.45  (0.43, 0.48) | – |  | 0.47  (0.44, 0.50) | – |
| Age, years | | | 0.05 |  | **< 0.01** | |
| <50 | 4141 | 0.43  (0.40, 0.46) |  |  | 0.41  (0.38, 0.43) |  |
| ≥50 | 2387 | 0.49  (0.45, 0.54) |  |  | 0.60  (0.55, 0.66) |  |
| Sex | | | 0.36 |  | 0.06 | |
| Male | 3274 | 0.46  (0.42,0.49) |  |  | 0.39  (0.36,0.42) |  |
| Female | 3254 | 0.45  (0.42,0.48) |  |  | 0.56  (0.52,0.61) |  |
| Education | | | 0.47 |  | 0.78 | |
| Less than high school | 1543 | 0.52  (0.46, 0.58) |  |  | 0.50  (0.44, 0.57) |  |
| High school | 1437 | 0.43  (0.38, 0.48) |  |  | 0.44  (0.39, 0.49) |  |
| College or  higher | 3548 | 0.45  (0.42, 0.48) |  |  | 0.47  (0.44, 0.51) |  |
| Race/ethnicity | | | **0.01** |  | **0.02** | |
| Hispanic | 1640 | 0.41  (0.37, 0.45) |  |  | 0.38  (0.34, 0.42) |  |
| Non-Hispanic  white | 3080 | 0.44  (0.41, 0.48) |  |  | 0.49  (0.45, 0.53) |  |
| Non-Hispanic  black | 1259 | 0.60  (0.55, 0.65) |  |  | 0.42  (0.39, 0.46) |  |
| Others Race | 549 | 0.47  (0.40, 0.56) |  |  | 0.57  (0.49, 0.66) |  |
| Family income–to-poverty ratio | | | 0.35 |  | 0.61 | |
| <1.30 | 1993 | 0.53  (0.48, 0.58) |  |  | 0.51  (0.46, 0.57) |  |
| 1.30-3.49 | 2392 | 0.43  (0.39, 0.48) |  |  | 0.47  (0.43, 0.52) |  |
| ≥3.50 | 2143 | 0.44  (0.41, 0.48) |  |  | 0.44  (0.41, 0.48) |  |
| LTPA | | | 0.49 |  | 0.19 | |
| Inactive | 2993 | 0.42  (0.39, 0.45) |  |  | 0.43  (0.40, 0.46) |  |
| Active | 3535 | 0.56  (0.50, 0.62) |  |  | 0.60  (0.54, 0.66) |  |
| BMI (kg/m^2^) | | | 0.17 |  | 0.55 | |
| <25 | 2042 | 0.42  (0.39, 0.46) |  |  | 0.50  (0.46, 0.54) |  |
| ≥25 | 4486 | 0.47  (0.44, 0.50) |  |  | 0.46  (0.43, 0.49) |  |
| Smoking status | | | **<0.01** |  | **0.02** | |
| Never | 3554 | 0.44  (0.41, 0.47) |  |  | 0.45  (0.42, 0.49) |  |
| Former | 1521 | 0.46  (0.41, 0.52) |  |  | 0.49  (0.44, 0.55) |  |
| Current | 1453 | 0.49  (0.45, 0.54) |  |  | 0.49  (0.45, 0.53) |  |
| Drinking status | | | 0.66 |  | 0.47 | |
| Never | 895 | 0.38  (0.33, 0.43) |  |  | 0.43  (0.37, 0.49) |  |
| Former | 1115 | 0.45  (0.40, 0.50) |  |  | 0.48  (0.42, 0.53) |  |
| Mild | 2078 | 0.47  (0.42, 0.51) |  |  | 0.49  (0.45, 0.54) |  |
| Moderate | 1039 | 0.46  (0.41, 0.52) |  |  | 0.50  (0.45, 0.55) |  |
| Heavy | 1401 | 0.47  (0.43, 0.52) |  |  | 0.44  (0.39, 0.49) |  |
| Hypertension | | | 0.47 |  | 0.33 | |
| No | 4049 | 0.45  (0.42, 0.48) |  |  | 0.45  (0.42, 0.49) |  |
| Yes | 2479 | 0.46  (0.43, 0.50) |  |  | 0.50  (0.47, 0.54) |  |
| DMs | | | 0.26 |  | 0.26 | |
| Normal | 5130 | 0.44  (0.41, 0.46) |  |  | 0.45  (0.42, 0.48) |  |
| Prediabetes | 476 | 0.56  (0.45, 0.70) |  |  | 0.59  (0.49, 0.71) |  |
| Diabetes | 922 | 0.55  (0.49, 0.61) |  |  | 0.57  (0.51, 0.64) |  |

The bold values mean statistical significance

Abbreviations: 3-PBA, 3-phenoxybenzoic acid; CI, confident interval; LTPA, leisure-time physical activity; BMI, body mass index; DMs, diabetes mellitus status.

Table S2 OR (95% CIs) for association between creatinine-corrected 3-PBA and OA in US adults.

| **Diseases** | Urinary creatinine-corrected 3-PBA, μg/g | | | | | *P* for trend |
| --- | --- | --- | --- | --- | --- | --- |
|  | Ln | Q1 | Q2 | Q3 | Q4 |  |
| **OA** | | | | | | 0.20 |
| Model 1 | **1.18**  **(1.11, 1.26)** | 1.00  (ref) | 1.23  (0.89, 1.69) | **1.43**  **(1.09, 1.86)** | **1.72**  **(1.27, 2.32)** |  |
| Model 2 | **1.09**  **(1.02, 1.17)** | 1.00  (ref) | 1.20  (0.84, 1.72) | 1.28  (0.94, 1.74) | 1.30  (0.94, 1.80) |  |
| Model 3 | **1.10**  **(1.01, 1.18)** | 1.00  (ref) | 1.19  (0.83, 1.71) | 1.27  (0.93, 1.73) | 1.31  (0.93, 1.85) |  |

The bold values mean statistical significance.

Model 1: No covariates were adjusted.

Model 2: Adjusted for age (continuous, years), education (less than high school, high school and college or higher), race/ethnicity (Hispanic, non-Hispanic white, non-Hispanic black, and others), sex (male, female) and family poverty-to-income ratio (<1.3, 1.3–3.5, >3.5).

Model 3: Adjusted for diet quality (continuous, HEI-2015 score), LTPA (inactive, active), smoking (never, former, and current), alcohol intake (never, former, mild, moderate, heavy), body mass index (<25 kg/m2, 25-29.9 kg/m2, ≥30 kg/m^2^), DMs (diabetes, prediabetes, normal) and hypertension (yes and no) plus variables in Model 2.

Abbreviation: OR, odds ratio; CIs, confidence intervals; OA, osteoarthritis 3-PBA, 3-phenoxybenzoic acid; Q, quartile.

Table S3 OR (95% CIs) for association between urinary creatinine-corrected 3-PBA and OA in subgroups.

| Group | Ln | Q1 | Q2 | Q3 | Q4 | *P* for trend | *P* for interaction |
| --- | --- | --- | --- | --- | --- | --- | --- |
| **Sex** | | | | | | | 0.11 |
| Male | 1.00 (0.88, 1.13) | 1.00 (ref) | 0.96 (0.53, 1.75) | 0.86 (0.54, 1.37) | 0.93 (0.54, 1.60) | 0.848 |  |
| Female | **1.17 (1.07, 1.30)** | 1.00 (ref) | 1.53 (0.97, 2.43) | **1.79 (1.17, 2.73)** | **1.84 (1.18, 2.87)** | 0.067 |  |
| **Age** | | | | | | | 0.44 |
| <50 | 1.06 (0.89, 1.27) | 1.00 (ref) | 0.79 (0.42, 1.51) | 1.02 (0.55, 1.91) | 0.93 (0.45, 1.93) | 0.979 |  |
| ≥50 | **1.10 (1.00, 1.21)** | 1.00 (ref) | 1.52 (0.98, 2.34) | **1.54 (1.06, 2.24)** | **1.54 (1.02, 2.32)** | 0.227 |  |
| **Race/ethnicity** | | | | | | | **<0.01** |
| Hispanic | 1.02 (0.86, 1.21) | 1.00 (ref) | 0.92 (0.40, 2.13) | 1.50 (0.71, 3.18) | 1.16 (0.49, 2.76) | 0.771 |  |
| Non-Hispanic white | **1.11 (1.03, 1.21)** | 1.00 (ref) | 1.34 (0.90, 2.01) | 1.33 (0.91, 1.93) | **1.50 (1.02, 2.19)** | 0.094 |  |
| Non-Hispanic black | 1.20 (0.99, 1.44) | 1.00 (ref) | 1.73 (0.73, 4.07) | 1.57 (0.74, 3.32) | 1.55 (0.73, 3.30) | 0.668 |  |
| Other Race | 0.61 (0.38, 0.96) | 1.00 (ref) | 0.12 (0.02, 0.75) | 0.31 (0.09, 1.11) | 0.09 (0.02, 0.57) | 0.047 |  |
| **BMI** | | | | | | | 0.99 |
| <25 | 1.04 (0.87, 1.23) | 1.00 (ref) | 1.29 (0.55, 3.05) | 1.28 (0.60, 2.76) | 1.22 (0.56, 2.66) | 0.861 |  |
| ≥25 | **1.11 (1.02, 1.21)** | 1.00 (ref) | 1.20 (0.81, 1.78) | 1.34 (0.91, 2.00) | 1.35 (0.93, 1.96) | 0.186 |  |
| **LTPA** | | | | | | | 0.39 |
| Active | **1.15 (1.00, 1.32)** | 1.00 (ref) | 1.09 (0.51, 2.35) | 1.49 (0.74, 3.02) | 1.71 (0.87, 3.35) | 0.057 |  |
| Inactive | 1.08 (0.98, 1.18) | 1.00 (ref) | 1.26 (0.85, 1.86) | 1.24 (0.86, 1.77) | 1.19 (0.82, 1.73) | 0.712 |  |
| **DMs** | | | | | | | 0.36 |
| Normal | **1.14 (1.04, 1.24)** | 1.00 (ref) | 1.15 (0.75, 1.76) | 1.18 (0.77, 1.81) | 1.45 (0.97, 2.17) | **0.049** |  |
| Prediabetes | 1.14 (0.90, 1.43) | 1.00 (ref) | 2.02 (0.70, 5.82) | 2.16 (0.62, 7.61) | 1.44 (0.47, 4.39) | 0.831 |  |
| Diabetes | 0.94 (0.79, 1.13) | 1.00 (ref) | 1.08 (0.43, 2.70) | 1.31 (0.60, 2.85) | 0.84 (0.38, 1.88) | 0.329 |  |
| **Hypertension** | | | | | | | 0.61 |
| No | 1.08 (0.96, 1.21) | 1.00 (ref) | 1.06 (0.59, 1.91) | 1.29 (0.73, 2.28) | 1.26 (0.72, 2.21) | 0.468 |  |
| Yes | 1.10 (0.99, 1.23) | 1.00 (ref) | 1.33 (0.85, 2.06) | 1.21 (0.80, 1.82) | 1.32 (0.83, 2.09) | 0.464 |  |

All models were adjusted for age, sex, race/ethnicity, education, family poverty-to-income ratio, HEI-2015, LTPA, smoking status, drink status, BMI, DMs and hypertension.

The bold values mean statistical significance.

Abbreviation: OR, odds ratio; CIs, confidence intervals; OA, osteoarthritis; 3-PBA, 3-phenoxybenzoic acid; Q, quartile; BMI, body mass index; LTPA, leisure-time physical activity; DMs, diabetes mellitus status.

National Health and Nutrition Examination Survey

(1999-2002/2007-2014)

Included for analyses (n=6,528)

Lack of sociodemographic information (n=710)

Availability of pyrethroids for adults aged over 20 years (n= 9565)

(n=)

Lack of information of osteoarthritis (n=826)

Lack of information of diet (n=485)

Lack of information of lifestyle and medical conditions (n= 1016)

Figure S1 Flow chart of participants included in final analysis.


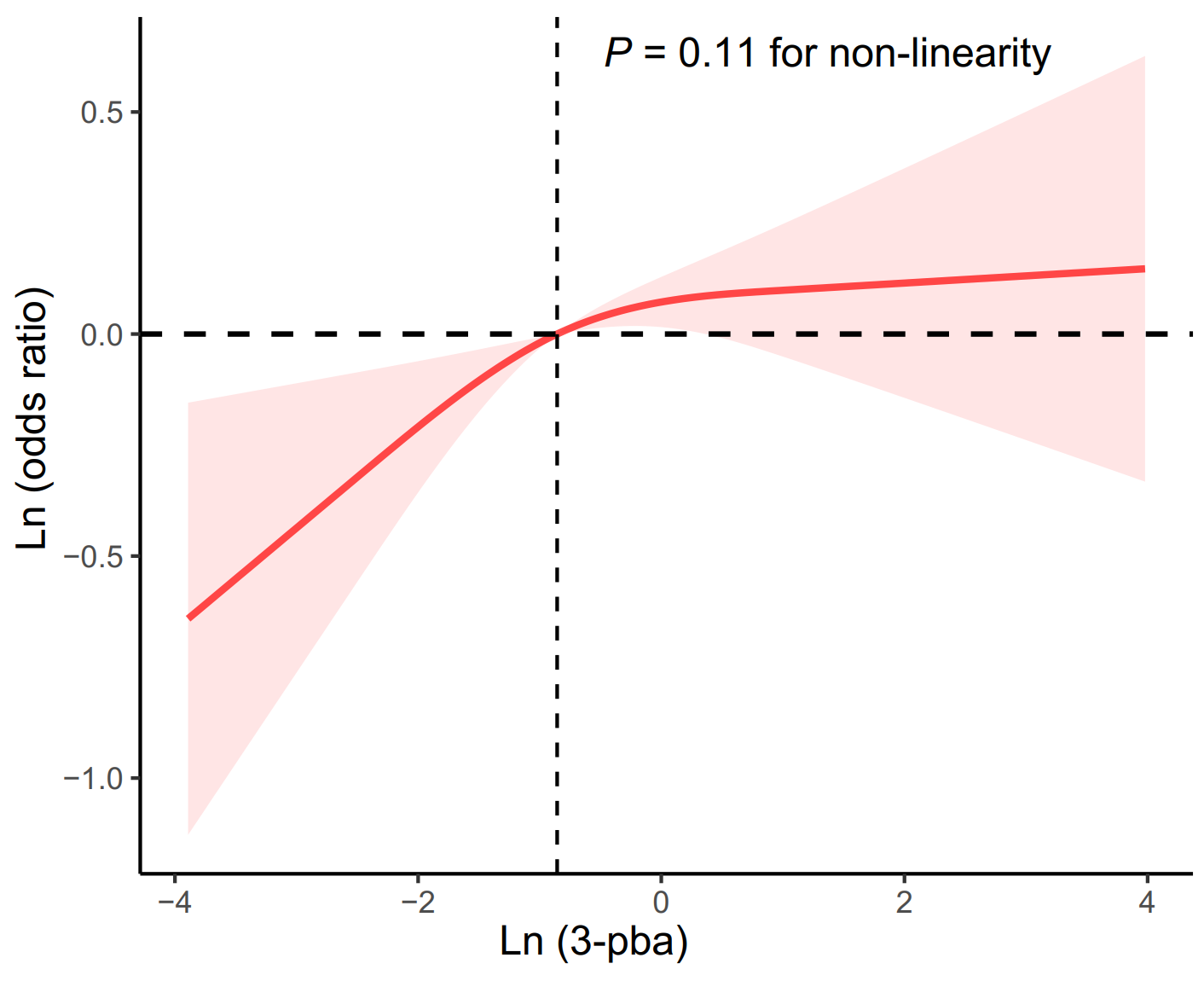


Figure S2 Dose-response association between urinary creatinine-corrected 3-PBA and OA.

Note. There exist linear associations between urinary creatinine-corrected 3-PBA and OA (*P* = 0.11 for non-linearity). The red lines and shaded areas represent the hazard ratios estimates and 95% CIs, respectively, relative to the reference level (dotted vertical lines). Models were adjusted for age, sex, race/ethnicity, education, family poverty-to-income ratio, HEI-2015, LTPA, smoking status, drink status, BMI, DMs and hypertension. Abbreviations: 3-PBA, 3-phenoxybenzoic acid; OA, osteoarthritis.
